# Trends and variation in data quality and availability on the European Union Clinical Trials Register: A cross-sectional study

**DOI:** 10.1177/17407745211073483

**Published:** 2022-02-11

**Authors:** Nicholas J DeVito, Ben Goldacre

**Affiliations:** Nuffield Department of Primary Care Health Sciences, University of Oxford, Oxford, UK

**Keywords:** European Union Clinical Trials Register, trial registration, transparency

## Abstract

**Background/Aims:**

The European Union Clinical Trials Register is a public facing portal containing information on trials of medicinal products conducted under the purview of the European Union regulatory system. As of September 2021, the registry holds information on over 40,000 trials. Given its distinct regulatory purpose, and results reporting requirements, the European Union Clinical Trials Register should be a valuable open-source hub for trial information. Past work examining the European Union Clinical Trials Register has suggested that data quality on the registry may be lacking. We therefore set out to examine the quality and availability of trial data on the registry with a focus on areas that fall under the authority of regulators in each European Union/European Economic Area country.

**Methods:**

Using data scraped from the full European Union Clinical Trials Register public dataset, we examined the extent of issues with three areas of trial data availability linked to European Union regulations. We examined whether there is evidence for missing registration of protocols in the public database, whether information on the completion of clinical trials is being made available and how often the results of trials are posted to the registry. We assessed each area overall, and examined variation between national regulators and over time.

**Results:**

Major issues with the availability of expected protocols and information on trial completion were focused in a few countries. Overall, when comparing enrolment countries from tabular results to available registrations, 26,932 of 31,118 (86.5%) expected protocols were available and 22 of 30 (73%) countries had over 90% of expected protocols available. The majority of missing protocols, totalling 2764 (66%), were from just three countries: France, Norway and Poland. Evidence for this issue is further supported by data on trends in new registrations by country over time. Low availability of data on trial completion is also most pronounced in a minority of countries, like Spain and the Netherlands, with consistent trends for missingness over time. Finally, overall results availability is substantially worse among the 23,623 trials with a single registered European Union protocol (*n* = 6259, 26.5%) compared to 13,897 of those taking place in multiple countries (*n* = 8423, 60.6%). Reporting for single-protocol trials was consistently low across both time and location.

**Conclusion:**

Deficiencies in the public availability of trial protocols, trial completion information and summary results complicate the utility of the European Union Clinical Trials Register for research, transparency and accountability efforts. Users of the registry would benefit from a more complete and accurate accounting of the European research environment via the official European Union registry. We recommend regulators at the national and pan-national level undertake routine audits of approved trials to ensure national-level issues are proactively and transparently identified, documented and addressed.

## Introduction

Registration of clinical trials in a publicly accessible registry helps ensure transparency and accountability in clinical research.^1–3^ Prospective and sufficiently detailed registrations allow public accounting of planned, ongoing and completed research; provide information on trial design and outcomes; and share these data in a convenient, open and accessible format.^4–9^ This is vital information for clinicians, researchers, health officials and the public.^10^ Trial registries are routinely used to survey the research agenda in a given area,^11–14^ as a check on potential reporting biases and research waste,^15–17^ and are recommended as a data source in evidence synthesis.^18^ There are persistent issues in ensuring registrations throughout science are used to evaluate reported studies;^19–22^ however, these concerns cannot begin to be properly addressed within clinical research unless the data on trial registries are accurate, timely and trustworthy. Without this confidence, registry data are easily dismissed.^23^ Issues with the provision of even basic required information about the conduct and results of studies undermine the value of registration.

Global requirements to register clinical trials proliferated throughout the early 2000s.^2,7,24^ The 2001 European Union (EU) Clinical Trial Directive required the establishment of a pan-European registry to house details of clinical trials of medicinal products.^25^ The EudraCT database, managed by the European Medicines Agency, was created in 2004 to hold trial information registered through the regulatory process in member states. The public facing repository of this information, the European Union Clinical Trials Register (EUCTR), would launch in 2011. Guidelines requiring the reporting of results of all completed trials directly to the registry were fully implemented in 2016.^26,27^ Trial sponsors must submit a clinical trial application in each country with planned enrolment in a given study. This application is essentially a standardised tabular protocol that is submitted to each national regulator (i.e. national competent authority) via the EudraCT system.^28,29^ Once regulatory and ethics approvals are in place and entered by the regulator, the country protocol is made public on the EUCTR under a single parent record.^30^ At the end of the trial, additional paperwork is filed to the regulator signalling its completion and this should then be noted in the public record. The regulator is then expected to follow up on the reporting of results within a year.^26,28,31^

If this system is functioning as intended, details of nearly all interventional medicinal research in Europe should be readily available on the public EUCTR. Evidence suggests that the flow of data through the EU regulatory system faces impediments. Audits of compliance with EU reporting guidelines have been hampered by outdated and inconsistent data.^32,33^ Trial status data on the EUCTR are often incongruent with matched registrations on ClinicalTrials.gov, the US registry.^34^ In 2019, administrative issues at the UK regulator led to protocol registrations not making it to the public EUCTR from the EudraCT backend.^35,36^ The European Medicines Agency has acknowledged issues with early EUCTR trial data through March 2011 (i.e. historical data) due to validation and institutional linkage issues and is working with national regulators ‘to ensure key data on the status of existing trials is complete’.^37,38^ Progress on this front, however, is undocumented and appears inconsistent. This analysis aims to comprehensively examine the aspects of registration on the EUCTR that are clearly linked to the regulatory process regarding the approval, completion and reporting of clinical trials. The findings will inform how and where national-level authorities may be failing to ensure trial information on the EUCTR is accurate, timely and complete. This can inform priorities for addressing gaps in the management of responsibilities under EU regulations and ensure the EUCTR is realising its value as a reliable data source for users.

## Methods

### Data collection

We used scraping software^39^ to collect data from each public country-level protocol on the EUCTR (i.e. all clinical trial applications) as of 1 December 2020. This was the last month in which new UK data were available on the EUCTR prior to leaving the EU and could therefore be compared to its European peers. As of 1 January 2021, UK sponsors may still add results to the EUCTR for existing registrations, but protocol adjustments, including updates on trial completion, are not possible and any ongoing protocols are tagged as no longer under the purview of the European Medicines Agency.^40^ Supplemental Box 1 shows an example of a master trial record on the EUCTR.

### Data extraction and definitions

The ‘National Competent Authority’ and ‘Trials Status’ fields were taken from the ‘Summary’ section of each protocol. For analyses involving time trends, the field ‘Date on which this record was first entered in the EudraCT database’ was used as this represents the date the regulator entered the trial record into the EudraCT database.^41^ This field was validated by comparison to the regulatory approval date listed in the registry for alignment (Supplemental Figure 4). Finally, the results status was identified by the presence of a ‘View Results’ link in a protocol.

### Study population

All trial records from an EU-regulated country as of December 2020 were included in our analysis. Trials from members of the European Economic Area (i.e. Norway, Iceland and Liechtenstein) also beholden to EU regulations are included in the dataset. Certain paediatric trials include non-EU protocols and these were excluded as they are not linked to any individual regulator and lack detailed information on trial completion by design. Phase 1 trials in healthy adults were excluded as these are not available on the public registry.^26^ Germany has two independent regulators that manage trial records and these were examined separately throughout unless otherwise noted. Information on each national competent authority as of December 2020 is available in Table 1.^42^

**Table 1.** Details of country-level regulators.

Country	Regulator	No. of trial protocols	First record entered
Austria	Austrian Federal Office for Safety in Health Care (BASG)	4146	16 July 2004
Belgium	Federal Agency for Medicines and Health Products (FAMHP)	5946	7 July 2004
Bulgaria	Bulgarian Drug Agency (BDA)	2007	2 February 2007
Croatia	Croatian Ministry of Health (MIZ)	401	24 January 2014
Cyprus	Ministry of Health Pharmaceutical Services (MoH PS)	5	24 February 2009
Czech Republic	State Institute for Drug Control (SUKL)	4304	24 June 2004
Denmark	Danish Medicines Agency (DKMA)	4069	10 August 2004
Estonia	Republic of Estonia Agency of Medicines (SAM)	1020	26 November 2004
Finland	Finnish Medicines Agency (FIMEA)	2533	26 May 2004
France	Agence Nationale de Sécurité du Médicament et des Produits de Santé (ANSM)	5852	21 June 2005
Germany	Federal Institute for Drugs and Medical Devices (BfArM)	8324	16 September 2004
Germany	Paul-Ehrlich Institut (PEI)	3193	10 September 2004
Greece	National Organization for Medicines (EOF)	1791	4 November 2005
Hungary	The National Institute of Pharmacy & Nutrition (OGYEI)	4473	15 June 2004
Iceland	Icelandic Medicines Agency (IMA)	133	7 September 2004
Ireland	Health Products Regulatory Authority (HPRA)	1169	18 June 2004
Italy	Italian Medicines Agency (AIFA)	7559	16 July 2004
Latvia	State Agency of Medicines of the Republic of Latvia (ZVA)	1079	3 August 2004
Liechtenstein	Amt für Gesundheit (AG)	0	NA
Lithuania	The State Medicines Control Agency (VVKT)	1237	22 June 2004
Luxembourg	Ministère de la Santé (MS)	8	26 July 2013
Malta	Medicines Authority (MDA)	18	10 October 2005
The Netherlands	Centrale Commisseei Mensfebonden Onderzoek (CCMO)	5692	16 March 2006
Norway	Norwegian Medicines Agency (NoMA)	683	25 May 2004
Poland	The Office for Registration of Medicinal Products, Medical Devices and Biocidal Products (URPL)	3242	29 March 2007
Portugal	Infarmed	1591	18 August 2005
Romania	National Agency for Medicines and Medical Devices (ANMDM)	239	14 July 2009
Slovakia	State Institute for Drug Control (SUKL)	1791	2 June 2004
Slovenia	Agency of the Republic of Slovenia for Medicinal Products and Medical Devices (JAZMP)	388	13 June 2005
Spain	Agencia Española de Medicamentos y Producto Sanitarios (AEMPS)	9566	14 June 2004
Sweden	Medical Products Agency (MPA)	3893	13 May 2004
The United Kingdom^a^	Medicines and Healthcare Products Regulatory Agency (MHRA)	10,975	1 July 2004

NA: not available.

a The United Kingdom has left the European Union and no longer participates in the European Medicines Agency system as of January 2021.

### Protocol availability

In order to examine the extent of missing protocol registrations, we selected all trials in the database that had results available in the EUCTR’s tabular format. This includes a standard data field indicating which countries enrolled participants in the trial. Using a custom web scraping programme,^43^ we extracted all enrolment countries from each trial with tabular results and compared them to the registered protocols associated with the trial registration. In practice, every EU location with confirmed enrolment in the results should have an associated public protocol. A country was only expected to have a protocol available if the trial start date (taken from the tabular results) was after the earliest available record entry date for that country on the entire registry (see Table 1) confirming an established link between the regulator and the European Medicines Agency. We report the expected versus actual protocol registrations based on the results information for each country and over time with reference to overall trends in new registrations.

### Quality of trial status and completion date fields

Each protocol on the EUCTR should reflect the current status of the trial and the date it completed in all countries once available.^28^ This date is officially called the ‘date of the global completion of the trial’ and is referred to here as the ‘global completion date’. Trial completion information is also entered in the ‘Results’ section, but this only exists for trials that have results and is not linked to official end of trial paperwork filed with a regulator and therefore was not considered in this analysis. We report the distribution of trial statuses and the availability of global completion dates in protocols overall, by country, and over time. Trials with conflicting trial completion information were also examined.

### Results availability

We separated all trials in our population into those that have a single EU protocol and those that have multiple protocols. Since reporting on the EUCTR occurs at the trial level and not the country level, for trials registered in a single country the responsibility for reporting follow-up falls solely within the remit of that country’s regulator. We report on the availability of results by country and over time with a focus on trials with a single protocol.

### Data and code availability

Data collection and analysis were performed in Python 3.8.1, and all data and code can be accessed via GitHub.^43,44^

## Results

As of 1 December 2020, the EUCTR contained 98,622 country-level protocols across 38,566 registered trial records since 2004. Removing all protocols from outside the direct regulatory purview of the European Medicines Agency leaves 97,227 protocols across 37,520 trials. Table 1 shows the total number of registered protocols and the earliest protocol entered on the registry from each regulator. The overall trend in new registrations and trials on the EUCTR is available in Supplemental Figure 1. Small countries with very low registered trial counts (Cyprus (*n* = 5), Luxembourg (*n* = 8), Malta (*n* = 18) and Liechtenstein (*n* = 0)) are not shown for some analyses as no meaningful trends could be established.

### Availability of protocols for countries with known enrolment

**Figure 1.**
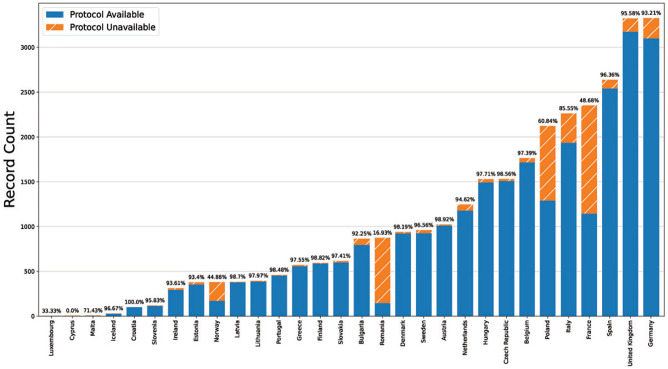
The number, and percentage, of protocols that should be publicly available for all trials with tabular results on the EUCTR. The actual protocols available for each country were compared to detailed tabular results, where available, that contain information on which specific countries enrolment was reported to have occurred.

**Figure 2.**
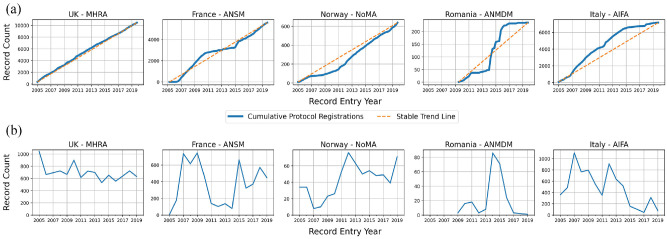
The image shows the (a) cumulative trend in new trials and (b) absolute trend in new trials for five countries. The United Kingdom is shown as a typical comparator country with a relatively stable rate of new trials, while the other four countries all have pronounced deviations in the registration of new protocols over time. The trends of all regulators are available in the Supplement.

When comparing registered protocols to known trial recruitment locations in the EU, availability was generally high. Of 31,118 expected protocols, 26,932 (86.5%) were available on the EUCTR and 22 of the 30 (73%) countries had more than 90% of expected protocols available while 17 of 30 (56%) had >95% available (Figure 1). Setting aside small countries with very few protocols expected, the lowest proportion of expected protocols was seen in France (48.7%), Norway (44.9%) and Romania (16.9%). Overall two-thirds (66.0%) of all missing protocols are from three countries: France, Norway and Poland. Italy (85.6%) was the only other high-output country with <90% availability. Evidence of these missing protocols can also be seen in the trends in new registrations by country. In most countries, between 2005 and 2019 (i.e. all full years in the dataset), new registrations remain relatively constant over time (e.g. the United Kingdom), while countries with many missing protocols inconsistently added new registrations (Figure 2, Supplemental Figures 2 and 3). Missing protocols were not limited to the ‘historic’ pre-2011 dataset as most were from records entered between 2012 and 2015 with a relatively low concentration of missing protocols from 2004 to 2009 (Supplemental Figure 5). According to the enrolment figures provided in the ‘Results’ section, missing protocols covered 1,265,740 enrolled trial participants.

### Completion status

**Figure 3.**
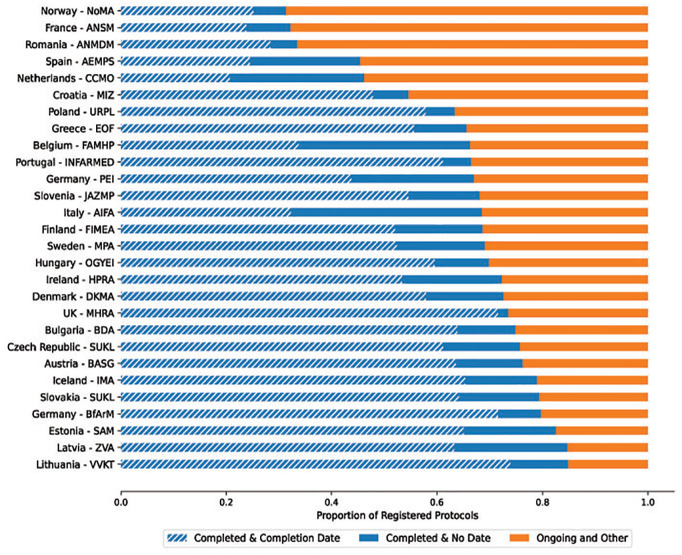
The proportion of trials in each regulator’s portfolio in a ‘Completed’ status (i.e. Completed or Prematurely Ended) is represented by the blue area and is broken down by those with a completion date (hashed blue) and without a completion date (solid blue). The number of ‘Ongoing and Other’ trials (in orange) in a status other than ‘Ongoing’ is nominal (see Supplement).

**Figure 4.**
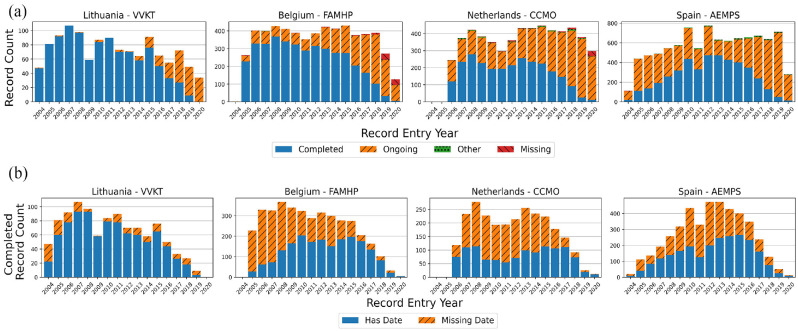
The image shows the (a) distribution of trial statuses of protocols first entered in each year and (b) trend in the proportion of completed protocols with a completion date across four selected countries. Lithuania represents the standard expected trend, while Belgium, the Netherlands and Spain represent issues in the availability of trial completion information.

Across all included trials on the EUCTR, 63,434 protocols (65.2%) are in a completed status; however, 21 of 28 (75%) regulators exceeded this mark across their registered protocols. Five regulators (Norway, France, Romania, Spain and the Netherlands) had <50% of their covered protocols in a completed status (Figure 3). There is a relatively consistent and expected pattern to the proportion of completed protocols in the database over time. From 2004 to 2013, the proportion of completed protocols among those first entered in that year ranges tightly from 76.6% to 83.5% before dropping off rapidly from 2014 as more recently registered trials remain ongoing (Supplemental Figure 6). Time trends by regulator generally follow this expected pattern (e.g. Lithuania) and deviations from this trend were minor, but consistent, in some countries (e.g. Belgium) and pronounced in others (e.g. the Netherlands, Spain) (Figure 4(a), Supplemental Figure 7). Of the 13,897 trial records with more than one country protocol, 4520 (32.5%) contain protocols in both a Completed and Ongoing status with older trials having the highest proportion of trials with conflicts (Supplemental Figure 8). For single-protocol trials, 6089 of 16,552 (36.8%) trials entered prior to 2015 are still in an ‘Ongoing’ status.

The overall availability of completion dates for completed protocols has remained consistent over time. Across the whole database, 75.6% of completed protocols have a global completion date available. From 2005 to 2018, the proportion of completed protocols first entered in that year that have a completion date never deviated far from this overall rate (range: 71.6%–79.2%) with an expected drop-off in 2019 and 2020 as recent studies may not have completed at all sites (Supplemental Figure 9). There is, however, variability among regulators. Of the 28 regulators examined, 20 (71.4%) exceeded the overall rate of global completion date availability and another three were within 2 percentage points (Supplemental Figure 10). The remaining five countries had a global completion date availability of <70%: Germany – Paul-Ehrlich Institut (65.3%), Spain (53.9%), Belgium (50.9%), Italy (47.2%) and the Netherlands (44.8%) (Figures 3 and 4(b)). Of the 8566 trials with multiple protocols and at least one global completion date, just 2002 (23.4%) have consistently provided the same global completion date across all protocols despite its linkage to required regulatory paperwork.

### Results availability

**Figure 5.**
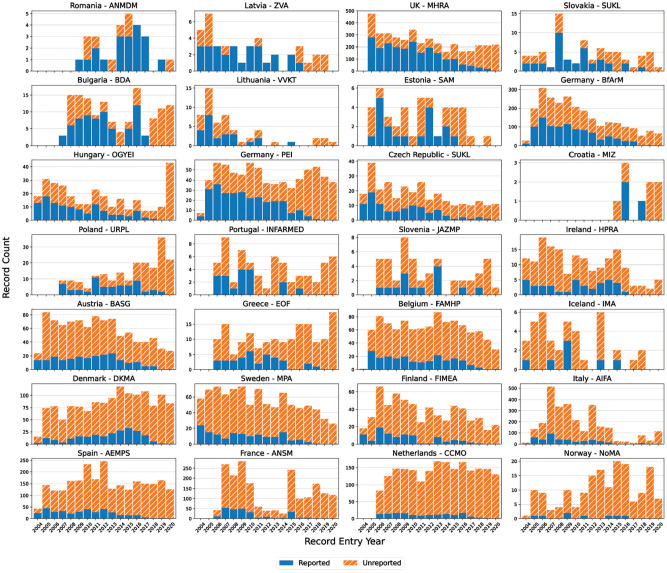
The trend in completed, single EU protocol trials that have results across responsible national regulators and over time. Regulators are ordered by percentage of all protocols reported. Since these trials fall under the oversight of a single regulator, there is no ambiguity in where enforcement of EU rules on trial reporting would sit.

Overall, the EUCTR contains 23,623 trials with a single EU protocol and 13,897 with multiple EU protocols. Results reporting for single-protocol trials were lower overall (26.5%) compared to multi-protocol trials (60.6%) and across all years in which protocols were submitted (Supplemental Figure 11). Only two regulators, Romania (70.4%) and Latvia (64.3%), exceeded a 60% reporting rate of single-protocol trials, but this represented just 69 reported trials. The next highest reporting country, the United Kingdom, reported 53.8% of 4057 trials. All high-research output peers of the United Kingdom fell below 40% of studies reported. Two countries had less than 10% of single-protocol trials reported: the Netherlands (7.5%) and Norway (4.7%) (Figure 5).

## Discussion

### Summary of results

There are notable gaps in some country’s data quality and availability on the EUCTR that have persisted over time. This study examined the extent of missing protocols, completion information and results. Issues with availability of public trial protocols and completion information are concentrated among a few very poorly performing countries, while results reporting deficiencies are more widespread.

### Strengths and weaknesses

This analysis covers all European trials on the EUCTR as of December 2020 and therefore provides a comprehensive and robust assessment of trends in registration and transparency practices throughout the continent. That said, this is a macro-level examination intended to spot systemic deviations. Some of this variation will be due to trial-level idiosyncrasies (e.g. trials with very long follow-up) that are out of the scope of this analysis to identify though the extent of the variation between countries is unlikely to be explained at the trial level. Our investigation of missing protocols is limited to trials with tabular results already on the EUCTR and is therefore only a proxy for the actual rate, and may understate the extent of the problem. The sample for the missing protocol analysis was also not random and may be representative of the sponsors and investigators most attentive to their trial records since they reported results. If single-protocol registrations, or multiple-protocol registrations without results, are missing at a higher rate than reported trials, this analysis may underestimate the extent of the issue.

The findings throughout this article are also not independent of one another and must be interpreted holistically. For instance, Romania has the highest percentage of single-protocol trials reported; however, it appears that the majority of Romanian trials are simply missing from the registry and cannot be checked for results. Finally, we only cover specific aspects of data quality on the EUCTR linked closely to regulator responsibilities and not the overall quality or accuracy of registered information about trial design and conduct.^45,46^

### Findings in context

Interpretation of our work is informed by prior studies examining clinical trial applications to major European national regulators using data directly from the regulators rather than the EUCTR.^47–49^ These show generally consistent trends in new applications over time. Perturbation in some trends, such as a decrease in new applications in Germany between 2009 and 2013, is mirrored in the EUCTR (Supplemental Figure 3: Germany – Bundesinstitut für Arzneimittel und Medizinprodukte); however, major deviations did not match. These studies consistently reported that France received between ∼700 and 1200 trial applications a year which is not aligned with EUCTR data.^48^ Dombernowsky and colleagues reported >800 trial applications per year to the French regulator in 2013 and 2014, while the EUCTR only contained 215 French protocols across both years. Another analysis in Nordic countries shows Sweden and Denmark broadly matched the EUCTR in the quantity of new applications (∼200–300 applications/year); however, between 400 and 500 applications were reported in Norway from 2004 to 2006, yet only 76 Norwegian protocols exist on the EUCTR for those years. It is unlikely that these large discrepancies would be the result of either large amounts of Phase 1 trial applications that are not public on the EUCTR or the vast majority of studies being rejected by regulators, and validates that our approach represents a genuine issue.

Issues with trial status are in accordance with prior work comparing the EUCTR and ClinicalTrials.gov, the US registry. Overall, 16.2% of trials available on both registries had a discrepant status, the vast majority of which had an ‘Ongoing’ status on the EUCTR but a ‘Completed’ status on ClinicalTrials.gov.^34^ This suggests a lower standard for data accuracy on completion in the EUCTR. Our results show some countries have unreasonably high numbers of currently ‘ongoing’ trials that started long ago, including the major trial hubs of Spain and the Netherlands. Viergever and colleagues examined the quality of a sample of registrations from 2012 taken from various registries, including a small number from the EUCTR. These quality assessments focused on the provision of contact information and descriptions of interventions and outcomes, and therefore did not overlap with the fields examined in this study. Point estimates for data quality in the EUCTR were mixed but with large confidence intervals due to the small sample. These findings suggest that additional quality issues may exist on the EUCTR beyond those described in this study, and further checks are warranted.^50^

Finally, we first encountered many of these potential data issues as part of our ongoing EU TrialsTracker work; however, this analysis represents the first attempt to formally document the extent of the issues.^32^ As of November 2021, the EU TrialsTracker, a monthly updating tracker of results reporting for completed trials on the EUCTR, showed 76.5% of verifiably completed trials had results. However, the conservative methodology used to identify when trials are due cannot properly assess trials with completion status and date issues. Transparency advocates have been similarly frustrated by these issues in their efforts to improve trial reporting throughout the EU.^51^ Here, we assessed results availability naive to any completion criteria and found data which suggests that the actual reporting percentage of completed trials would be lower with more accurate completion data. Single-protocol trials make up 63% of all registered trials but just 43% of all results on the registry and many of these lack required trial completion information. In addition, our EU TrialsTracker work noted large reporting discrepancies between industry and non-industry sponsors, as well as large and small sponsors.^32,52^ These discrepancies likely account for much of the observed gap in reporting between single-protocol and multi-protocol trials given the frequency of multi-national industry–funded trials.

### Policy implications and interpretation

EU countries are a major source of medical research globally and their registration scheme is tied directly to national and EU regulations.^53^ Documenting basic trial details should be ensured through the routine regulatory process.^25,26,54^ Providing accurate data to the EUCTR fulfils both ethical and legal obligations. As a primary member of the World Health Organization International Clinical Trial Registration Platform, the EUCTR commits to ‘make all reasonable efforts to ensure that the data registered is complete, meaningful, and accurate’.^55^ The issues found in this analysis ultimately frustrate efforts to use the EUCTR as a canonical data source for trial information in Europe, despite its considerable promise. Conflicting information on trial completion and missing results creates additional burden for users in analysing, interpreting and acting on data from the registry. Missing public registrations may also complicate publication for researchers who rely on the EUCTR to satisfy journal requirements for prospective registration.^56^ When regulators cannot ensure the most basic of required record-keeping tasks are completed, it undermines trust in their authority and confidence that due diligence of regulated research is being performed.

The information pipeline from sponsors, to national regulators, to the EUCTR appears to have been disrupted across multiple countries. These include major European research hubs like France, Spain and the Netherlands, which is now home to the European Medicines Agency. While our findings cannot alone determine whether these issues primarily originate with the regulators, sponsors or some combination of both, prior public reports are informative. As described above, data on trends in clinical trial applications to various national regulators do not support the year-over-year fluctuations in new trials on the EUCTR seen in some countries. Other anomalies also exist; for instance, it would appear impossible that Romania – the sixth most populous country in the EU – only approved 239 clinical trials since entering the Union in 2007. A search of ClinicalTrials.gov for interventional drug trials in Romania over the same period returns >1500 results (https://tinyurl.com/rwjxc3n5). In the United Kingdom, delays in public protocol availability were caused by lack of administrative staff for data entry. Once these staffing issues were resolved, protocol availability improved as did the updating of trial completion information.^35,36,57^ In totality, it appears protocol availability issues largely originate at the regulator level when they fail to act accordingly on required information provided by sponsors.

While failure to provide results information ultimately falls to trial sponsor, regulators can play a more active role in promoting and following up on reporting as envisioned by the guidelines.^26^ The Austrian regulator conducted sponsor outreach concerning the results reporting requirements and has seen subsequent increases in results submissions.^58^ The European Medicines Agency^59^ has also conducted proactive outreach to remind sponsors of their responsibility to report, but national regulators may be better positioned for outreach within the local regulatory context. Issues with the provision of completion information are more complicated to understand from afar. End of trial documentation is required as part of the EU regulatory process, but it is difficult to know whether the gaps originate with sponsors failing to submit the necessary paperwork or regulators failing to act on paperwork after it is provided, although either would be concerning. In the best-case scenario, the proper paperwork is archived with regulators but has simply not been acted upon. Addressing these issues could be rectified through concerted record-keeping and data-entry efforts. Regular audit cycles from both national regulators and the European Medicines Agency, similar to the analysis presented here, would allow timely follow-up of missing information from either sponsors or national regulators. The shared code for this analysis could provide a template to make this process trivial. The Heads of Medicines Agencies organisation, a network of EU regulatory leadership, may be an effective partner for coordinating improvement and sharing best practices between regulators. The Heads of Medicines Agencies has recently announced plans to further encourage reporting to the EUCTR in response to external pressure.^60,61^ The UK Medicines & Healthcare products Regulatory Agency’s work to address issues has been documented and could aid other national authorities in understanding how their processes could be improved.^62^ Ultimately, flexibility in working with sponsors outside rigid bureaucratic rules, especially in rectifying data from very old trials, may be warranted to improve data quality on the EUCTR.

A new EU trial portal is set to launch in January 2022, but the EUCTR should not be neglected as an important source of clinical trial information. The corpus of registered trials from 2004 through the 2023 phase-out of new registrations on the EUCTR contains information on thousands of trials of treatments in wide use today.^63^ While the new portal promises more streamlined registration and approval processes across EU locations, national regulators will still play an important oversight role.^64^ Individual countries will also be empowered to sanction non-compliant sponsors.^54^ Key learnings from the implementation of the current clinical trial regulations should inform staffing needs and internal processes moving forward and ensure adequate resourcing for monitoring of data quality and results reporting for regulated trials.

## Conclusion

There are persistent and notable gaps in the quality and completeness of trial data on the EUCTR. The public dataset appears to, frequently, be missing registrations, trial completion information and results, especially from certain countries. The processes that guide the collection and dissemination of these data are embedded in a clear regulatory structure so their apparent failure is concerning and undermines confidence in both the EUCTR as a data source and the soundness of the overall regulatory process. Users of the EUCTR, and those patients and citizens impacted by the decision making it informs, would benefit from a more complete and accurate accounting of the European clinical research environment. Steps should be taken to ensure issues at the level of national regulators are proactively and transparently identified, documented and addressed.

## Supplemental Material

sj-docx-1-ctj-10.1177_17407745211073483 – Supplemental material for Trends and variation in data quality and availability on the European Union Clinical Trials Register: A cross-sectional study Click here for additional data file. Supplemental material, sj-docx-1-ctj-10.1177_17407745211073483 for Trends and variation in data quality and availability on the European Union Clinical Trials Register: A cross-sectional study by Nicholas J DeVito and Ben Goldacre in Clinical Trials
