# Supplementary material for: Trends and variation in data quality and availability on the European Union Clinical Trials Register: A cross-sectional study
Source: Clin Trials. 2022 Feb 11;19(2):172–83. doi: 10.1177/17407745211073483 (PMC9036151; doi:10.1177/17407745211073483)
Supplement: sj-docx-1-ctj-10.1177_17407745211073483 – Supplemental material for Trends and variation in data quality and availability on the European Union Clinical Trials Register: A cross-sectional study [file sj-docx-1-ctj-10.1177_17407745211073483.docx]

**Supplementary Information: DeVito & Goldacre**

[**Supplemental Box 1: Example of an EUCTR Master Trial Record**](#_idhkuhd6qds2) **2**

[**Supplemental Figure 1: Overall Registration Trends**](#_fg2ny5hbq7s) **3**

[**Supplemental Figure 2: Cumulative Trends in New Trials by Country**](#_azd6unz96dfj) **4**

[**Supplemental Figure 3: Annual Trend in New Trials Registrations From Each Regulator**](#_rt8po0sgww7c) **5**

[**Supplemental Figure 4: Comparison of Available Key Regulatory Dates**](#_ecpw1r4wma5y) **6**

[**Supplemental Figure 5: Missing Protocols by Trial Entry Year**](#_ves54284d16n) **7**

[**Supplemental Figure 6: Trial Status of Registered Protocols by Record Entry Year**](#_w8s9vcv7l3so) **8**

[**Supplemental Figure 7: Trial Status by Country Regulator**](#_tlp5qcvmt6ik) **9**

[**Supplemental Figure 8: Registered Protocols in a Completed Status**](#_kitr0l7jw1pc) **10**

[**Supplemental Figure 9: Availability of Completion Date for Completed Protocols**](#_5j1eev4sapjx) **11**

[**Supplemental Figure 10: Trends in Completion Date Availability by National Regulator**](#_79rwffud4nv) **12**

[**Supplemental Figure 11: Results Availability by Year**](#_47kxk04d4bqr) **13**

# Supplemental Box 1: Example of an EUCTR Master Trial Record

| 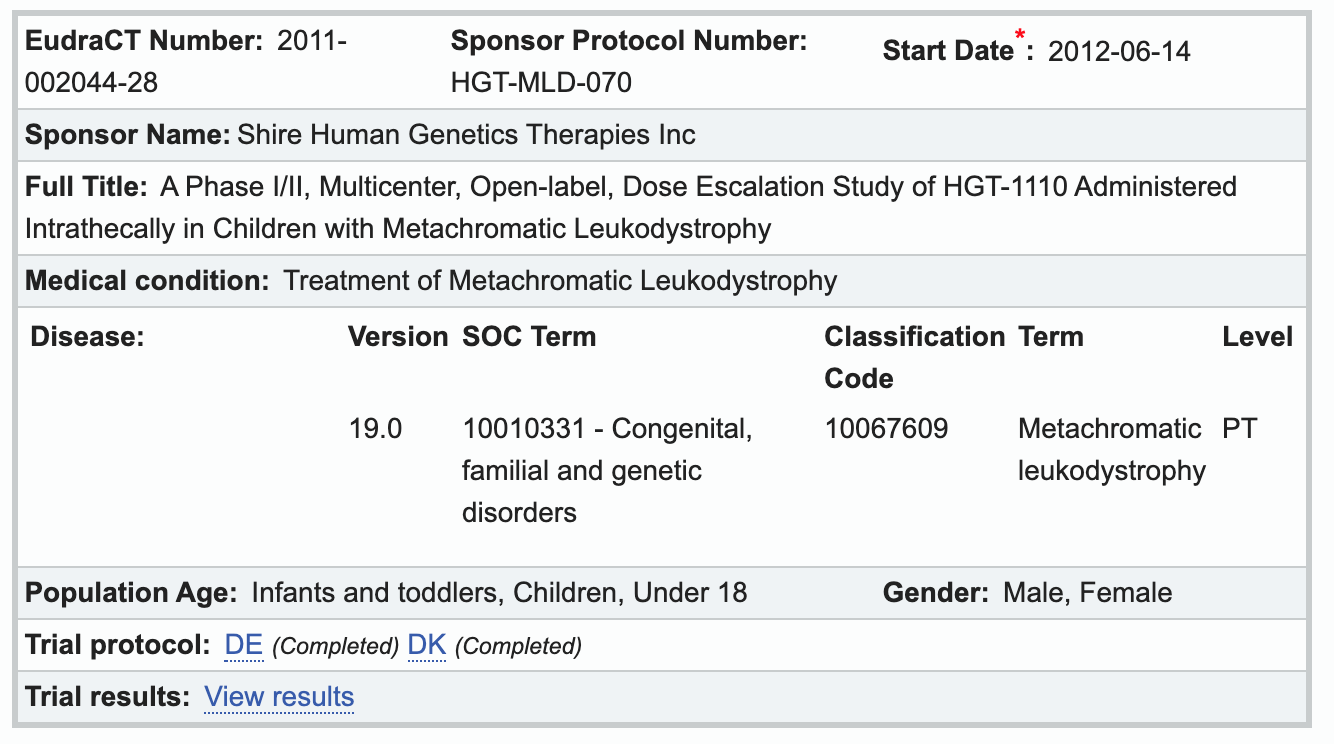 |
| --- |

***Supplemental Box 1:*** *This is the master trial record displayed for a public registration on the EUCTR. Each protocol can be accessed via the links in the “Trial Protocol” section and results, if available via the “View Results” link.*

#

# Supplemental Figure 1: Overall Registration Trends


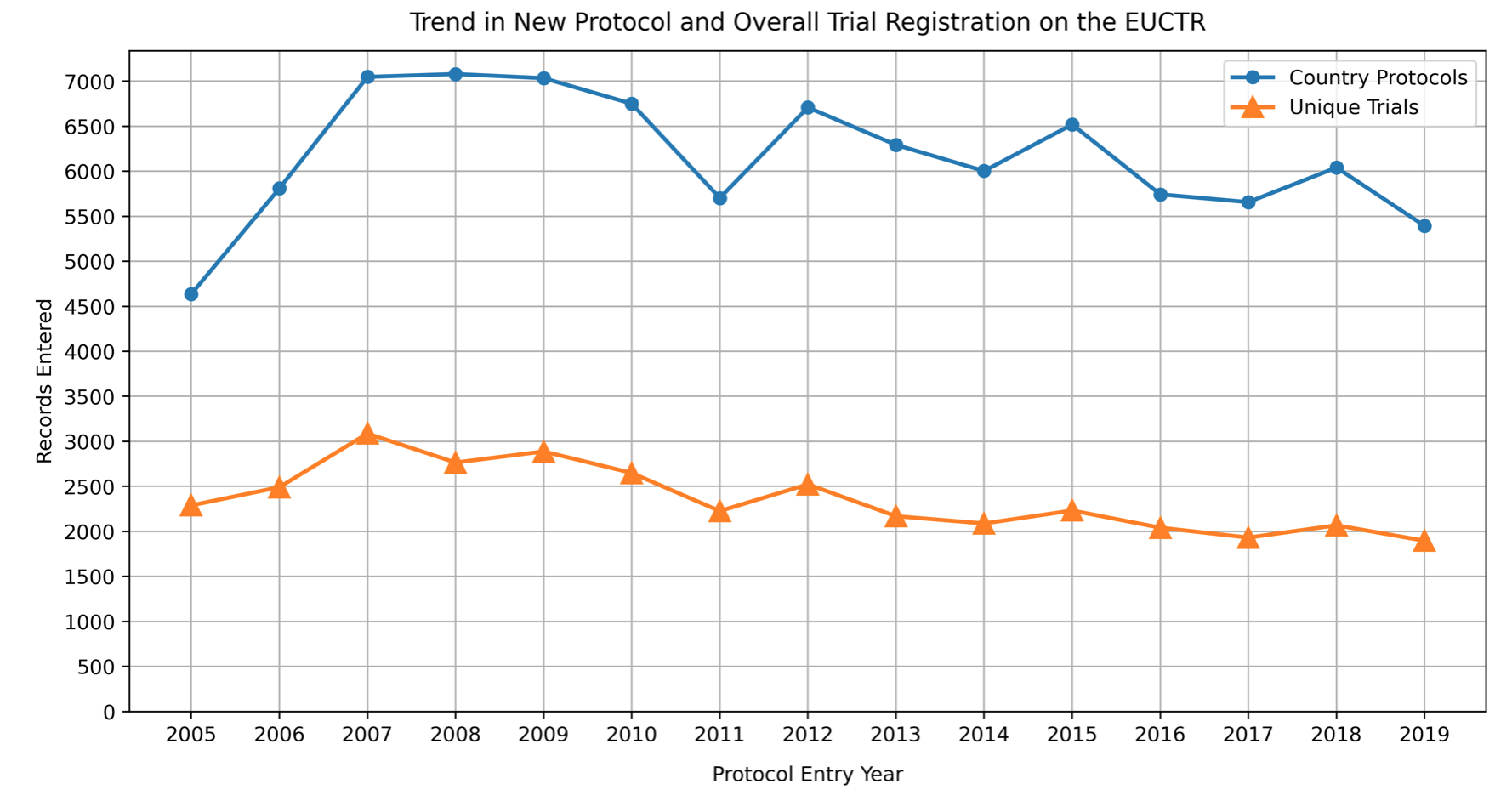


***Supplemental Figure 1:*** *The overall trend in number of new EU/EEA protocol and full-trial registrations for all years with full data (i.e., excluding 2004 and 2020). A single trial registration on the EUCTR is made up of individual EU/EEA protocols for countries with current or planned recruitment.*

# Supplemental Figure 2: Cumulative Trends in New Trials by Country

**
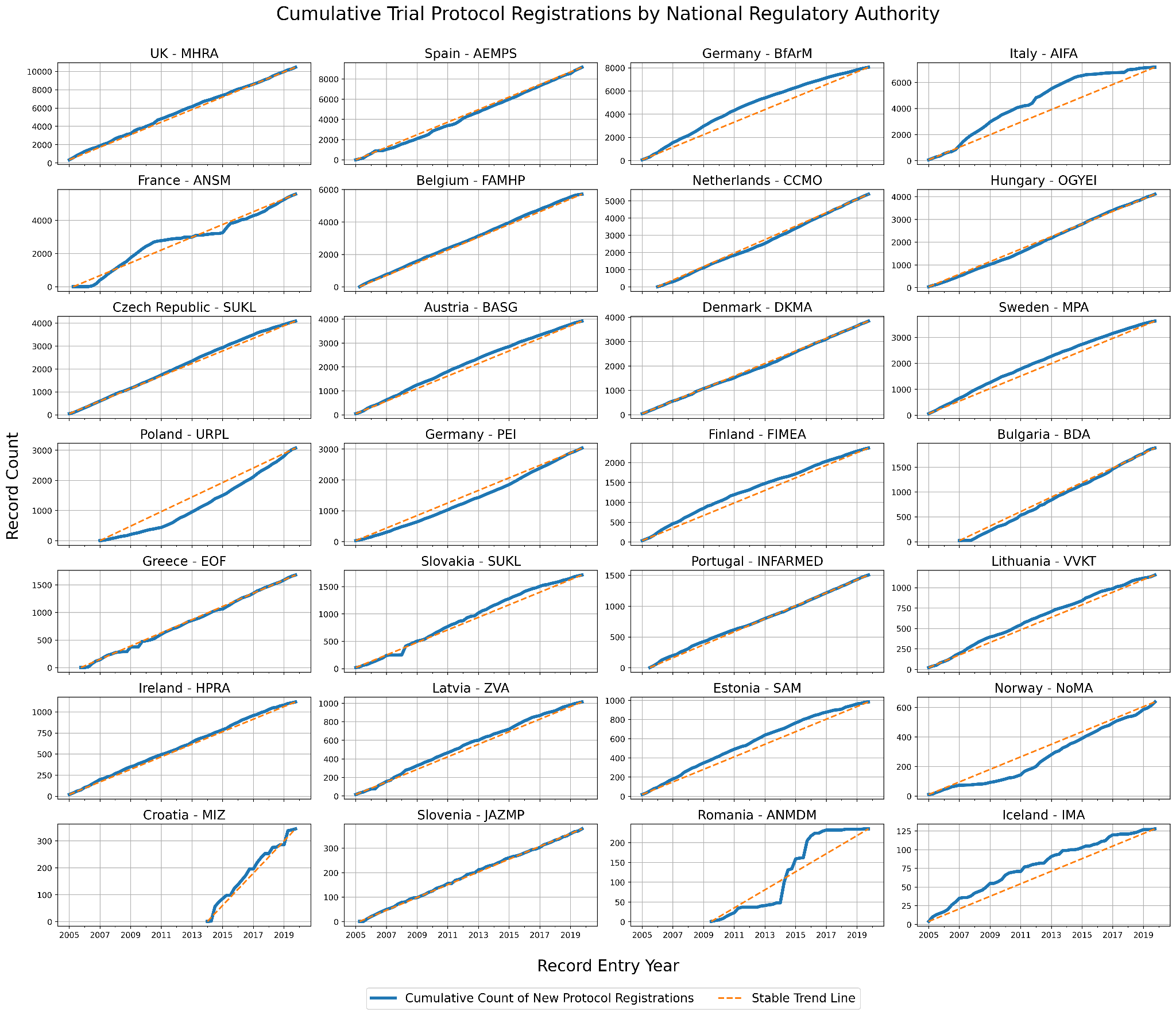
**

***Supplemental Figure 2:*** *The cumulative trend in new protocol registrations by each regulator. The dotted orange line represents what a stable trend in new registrations over time would look like. Data here was aggregated by quarter for more precise visualisation of the trend over time.*

# Supplemental Figure 3: Annual Trend in New Trials Registrations From Each Regulator

**
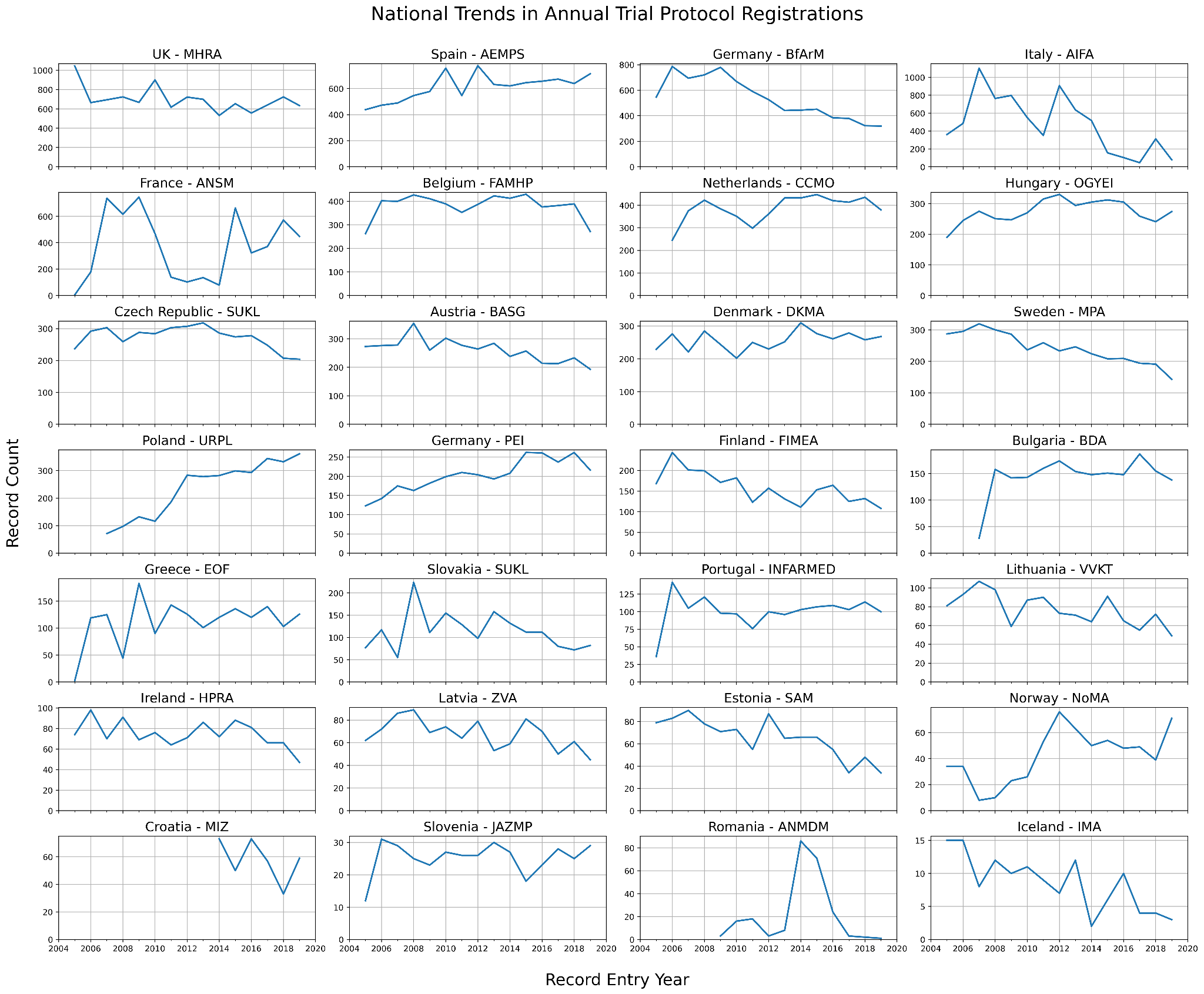
**

# Supplemental Figure 4: Comparison of Available Key Regulatory Dates


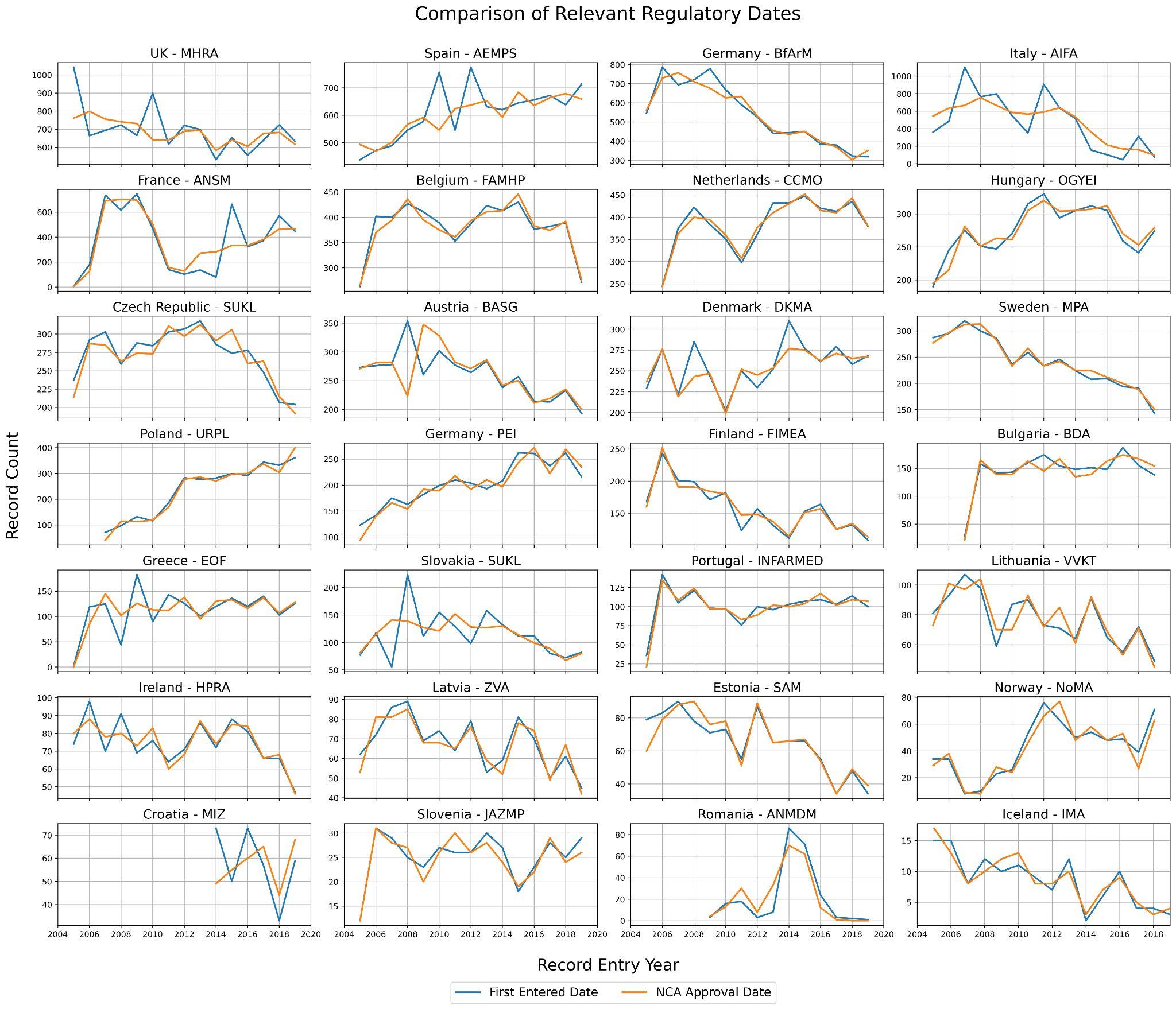


***Supplemental Figure 4:*** *The annual trend in new protocol registrations by each regulator. Here we compare the trend using the field “Date on which this record was first entered in the EudraCT database”, which was used throughout the manuscript, to the “Date of Competent Authority Decision”. While regulatory approval dates generally represent a smoother trend, they match very closely over time suggesting there would be no variation in overall conclusions based on choosing one date over the other.*

# Supplemental Figure 5: Missing Protocols by Trial Entry Year


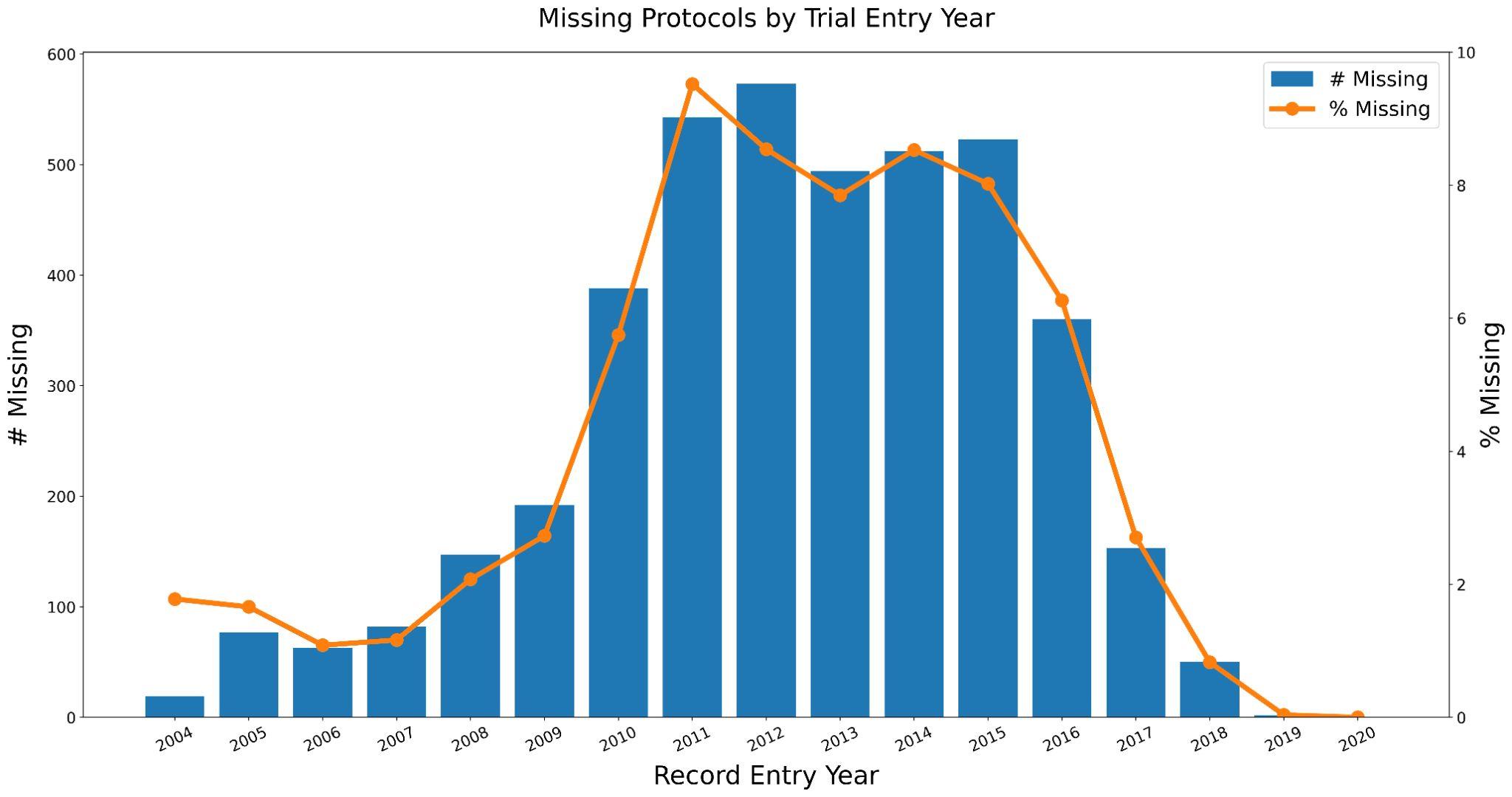


***Supplemental Figure 5:*** *The bars represent the total number of missing protocols by the year in which the earliest public protocol was entered for the parent trial registration. The line represents the missing protocols for trials first entered in that year as a percentage of all publicly available protocols first entered in that year.*

# Supplemental Figure 6: Trial Status of Registered Protocols by Record Entry Year


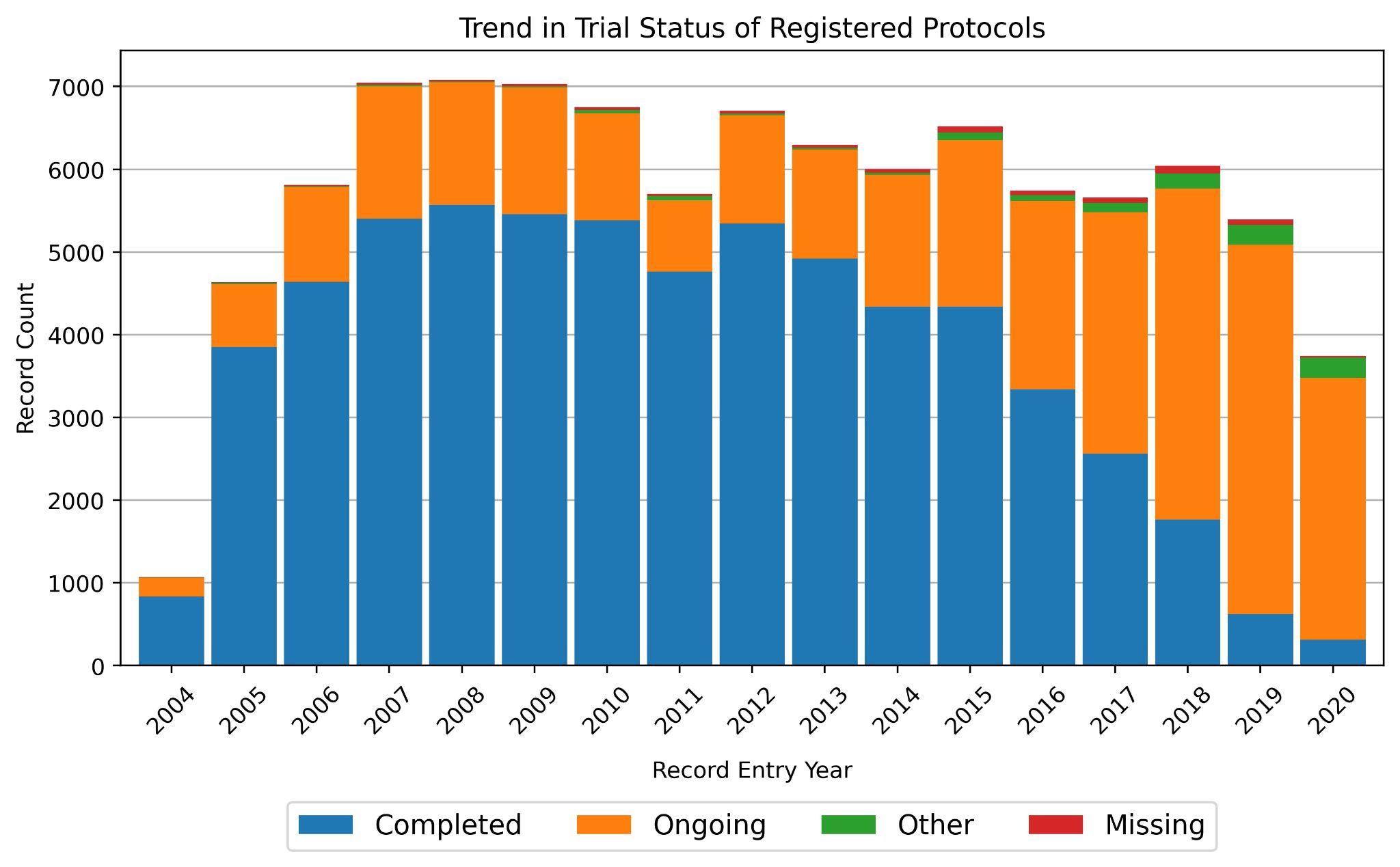


***Supplemental Figure 6:*** *This graph shows the distribution of trial status for all protocols entered in each year. As expected the proportion of trials in a “Completed” status decreases over time as more recent trials are still ongoing.*

# Supplemental Figure 7: Trial Status by Country Regulator

**
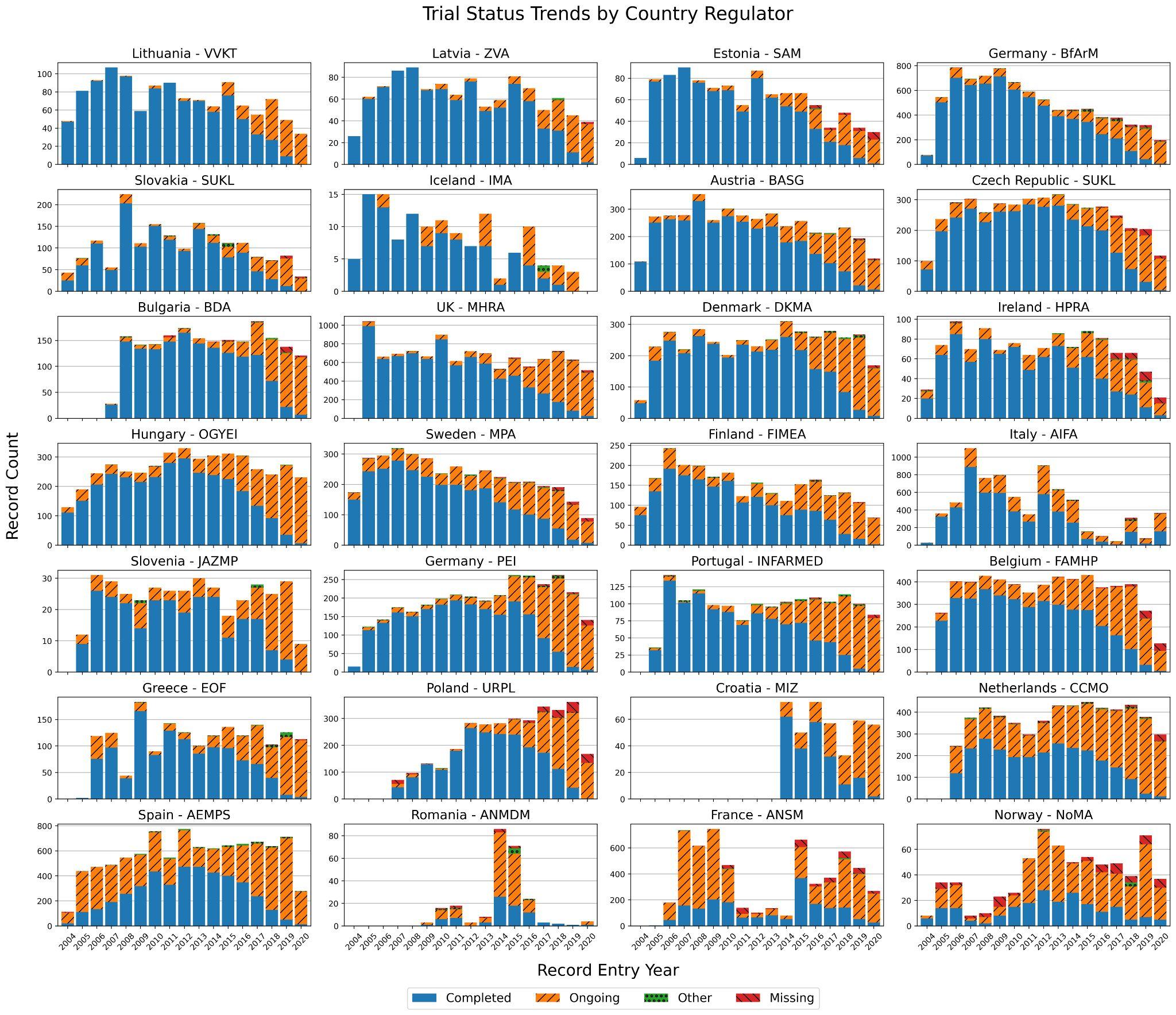
**

***Supplemental Figure 7:*** *The typical trend in completion date availability can be seen across most countries.*

#

# Supplemental Figure 8: Trials with Conflicting Trial Status

**
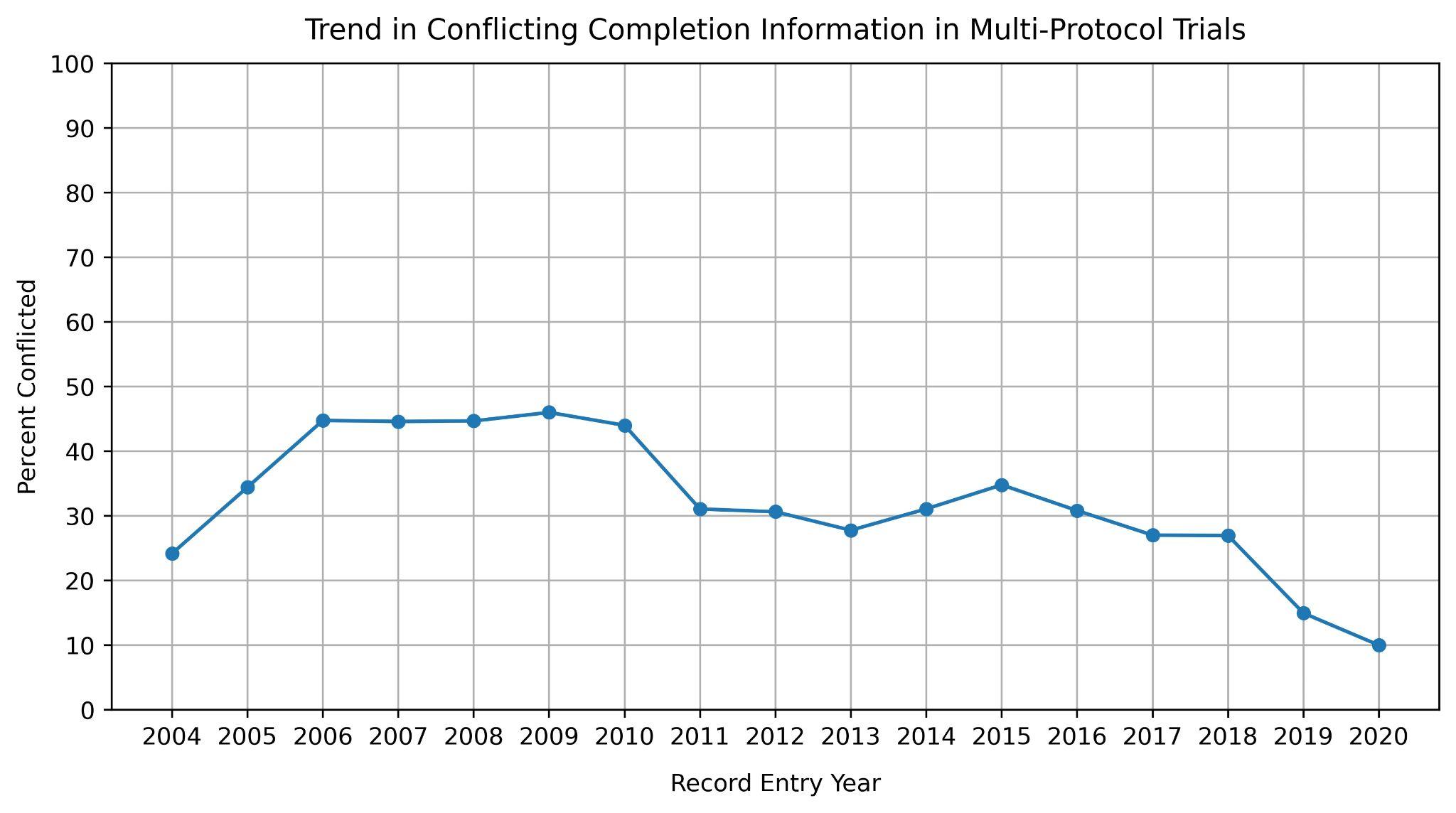
**

***Supplemental Figure 8:*** *The record entry year represents the latest record entry date across all protocols for a given trial. The percent of all trials from a given year currently have conflicting status information between protocols is shown.*

# Supplemental Figure 9: Availability of Completion Date for Completed Protocols


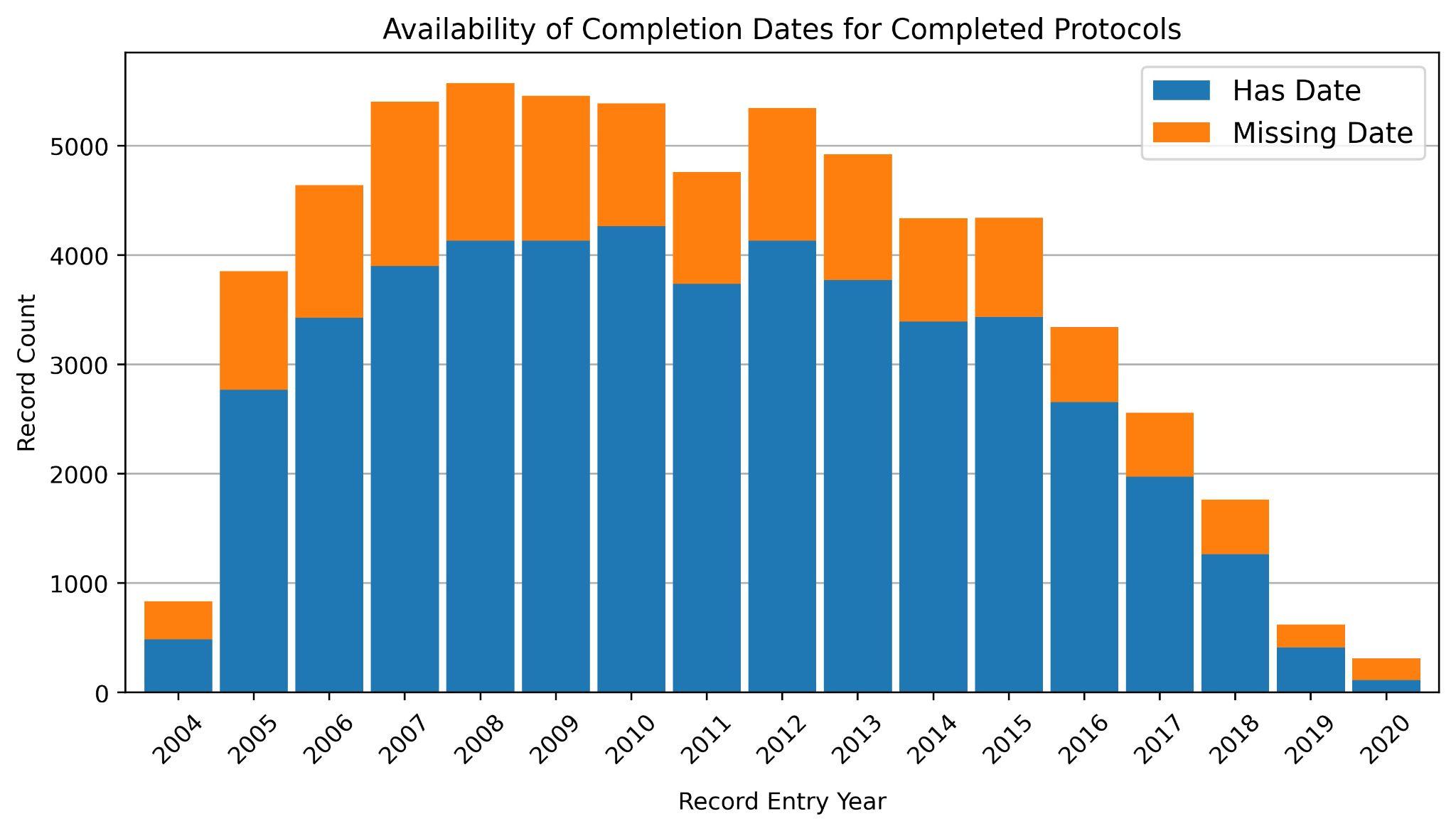


***Supplemental Figure 9:*** *The overall trend in completion date availability by record entry year. Missing competition dates persist in the data beyond the “historical”.*

# Supplemental Figure 10: Trends in Completion Date Availability by National Regulator

**
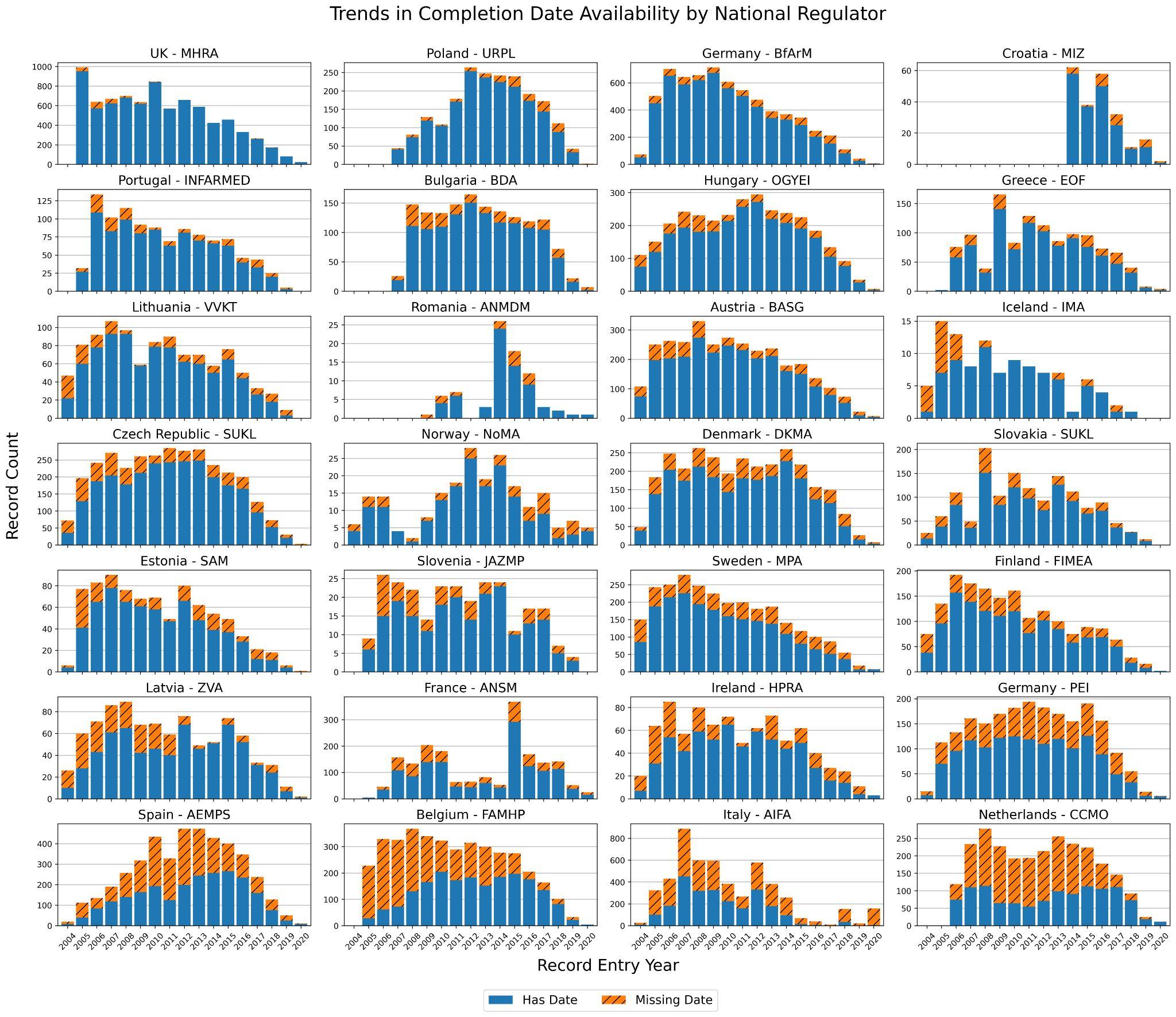
**

***Supplemental Figure 9:*** *The availability of completion dates among all completed protocols is shown for each regulator with poor performance concentrated among a few sponsors.*

# Supplemental Figure 11: Results Availability by Year

**
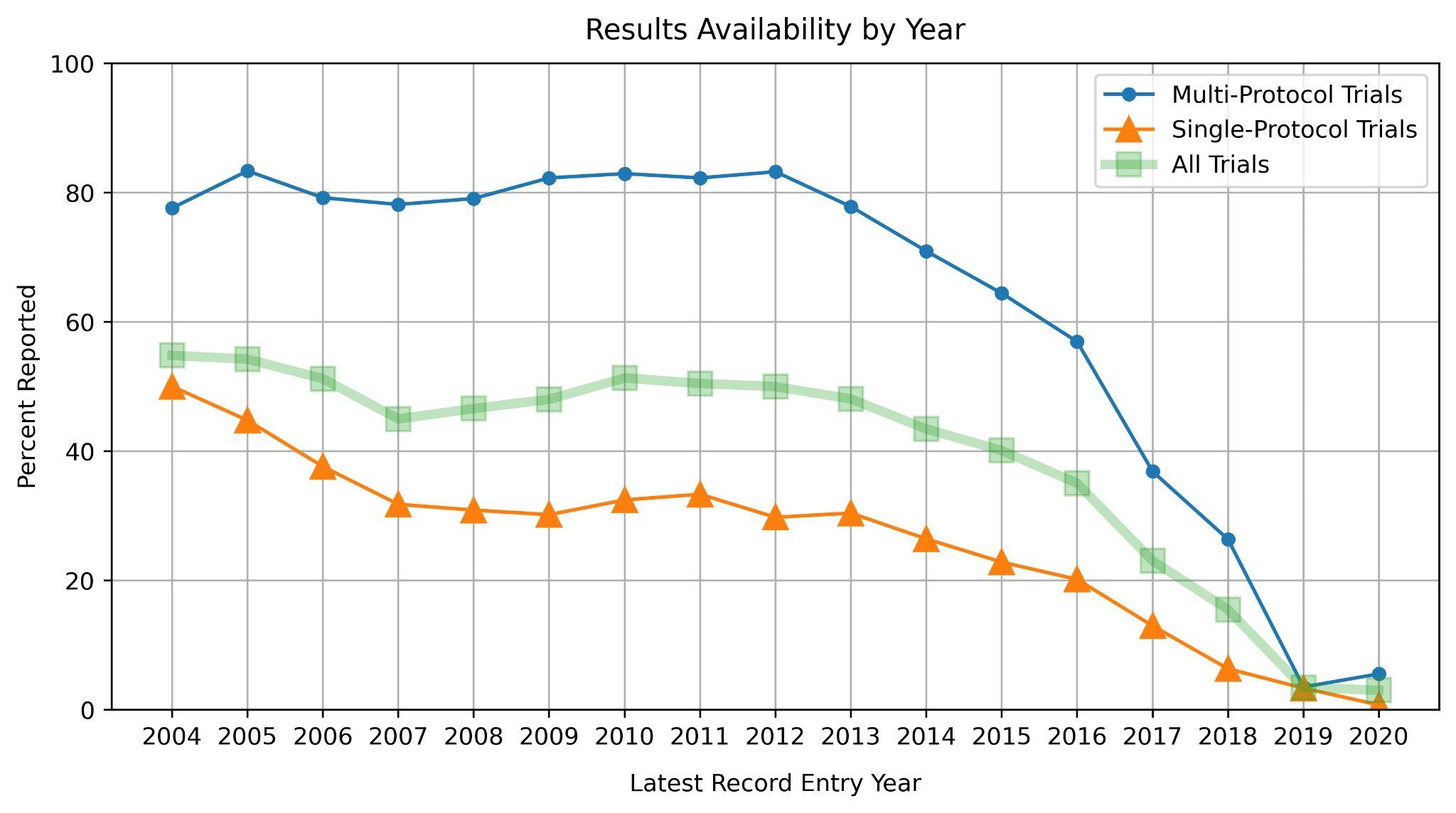
**

***Supplemental Figure 11:*** *The percent of trials that have an associated results report by year of the latest protocol-record entry date for a given trial. Data is split for trials with a single protocol and for those with multiple included protocols. The drop-off in results for more recent trials is expected as many of these either will have only recently completed or are still ongoing.*
